# Chemokine receptor expression defines a trajectory from monocytes to mature macrophages in the lung

**DOI:** 10.1093/jleuko/qiag059

**Published:** 2026-05-06

**Authors:** Heather Mathie, Laura Medina-Ruiz, Fabian Schuette, Heba Halawa, Zuzanna Pocalun, Elise Pitmon, John Cole, Marieke Pingen, Gerard J Graham

**Affiliations:** Chemokine Research Group, School of Infection and Immunity, College of Medical, Veterinary and Life Sciences, University of Glasgow, 120 University Place, Glasgow G12 8TA, United Kingdom; Chemokine Research Group, School of Infection and Immunity, College of Medical, Veterinary and Life Sciences, University of Glasgow, 120 University Place, Glasgow G12 8TA, United Kingdom; Chemokine Research Group, School of Infection and Immunity, College of Medical, Veterinary and Life Sciences, University of Glasgow, 120 University Place, Glasgow G12 8TA, United Kingdom; Chemokine Research Group, School of Infection and Immunity, College of Medical, Veterinary and Life Sciences, University of Glasgow, 120 University Place, Glasgow G12 8TA, United Kingdom; Chemokine Research Group, School of Infection and Immunity, College of Medical, Veterinary and Life Sciences, University of Glasgow, 120 University Place, Glasgow G12 8TA, United Kingdom; Chemokine Research Group, School of Infection and Immunity, College of Medical, Veterinary and Life Sciences, University of Glasgow, 120 University Place, Glasgow G12 8TA, United Kingdom; Chemokine Research Group, School of Infection and Immunity, College of Medical, Veterinary and Life Sciences, University of Glasgow, 120 University Place, Glasgow G12 8TA, United Kingdom; Chemokine Research Group, School of Infection and Immunity, College of Medical, Veterinary and Life Sciences, University of Glasgow, 120 University Place, Glasgow G12 8TA, United Kingdom; Chemokine Research Group, School of Infection and Immunity, College of Medical, Veterinary and Life Sciences, University of Glasgow, 120 University Place, Glasgow G12 8TA, United Kingdom

**Keywords:** chemokine receptors, inflammation, lung, macrophages

## Abstract

CCR1, CCR2, and CCR5 direct recruitment of monocytes and macrophages in inflammation. However, the discrete role for each receptor in monocyte/macrophage biology remains poorly understood, with previous reports citing receptor redundancy. Using transcriptomic approaches to examine inflammatory chemokine receptor expression on lung interstitial macrophage populations, we demonstrate that interstitial macrophages can be divided into 3 distinct subsets, each of which express specific patterns of chemokine receptors, and that there are dynamic changes in chemokine receptor expression as macrophages differentiate from monocytes in the lung. Furthermore, macrophages expressing different combinations of chemokine receptors are transcriptionally distinct, suggesting nonredundant functions for CCR1, CCR2, and CCR5. Finally, we examined changes in macrophage chemokine receptor expression in vitro after treatment with varied Toll-like receptor ligands and show that CCR1 is specifically increased in response to bacterial but not viral ligands. Our data provide compelling evidence that macrophage chemokine receptor expression is not redundant, but rather is specific and malleable in response to discrete inflammatory stimuli.

## Teaser

Interstitial macrophages exist in three transcriptionally distinct subsets and alter inflammatory chemokine receptor expression as they differentiate from monocytes in the lung.

## 1. Introduction

Monocyte-derived macrophages are an essential component of the inflammatory response and are implicated in pathogen and cellular debris removal, and wound healing.^1^ The recruitment of monocytes to inflamed sites, and the interstitial movement of their macrophage progeny within the inflamed tissue, are regulated mainly by members of the chemokine family.^2^ Chemokines are characterized by a conserved cysteine motif and divided into CC, CXC, XC, and CX3C subfamilies accordingly.^3,4^ They mediate their effects through cognate 7-transmembrane spanning receptors, which are named according to the subfamily of chemokine to which they bind (i.e. CCR, CXCR, XCR, and CX3CR).^5^ Broadly speaking, chemokines, and their receptors, can be defined as being inflammatory or homeostatic according to the contexts in which they function.^4^ Monocyte and macrophage dynamics during the inflammatory response are predominantly controlled by inflammatory CC chemokines and their cognate receptors.

We are interested in defining chemokine receptor involvement in monocyte and macrophage dynamics at inflamed sites. Literature in this area is confusing, with reports suggesting significant redundancy of chemokine receptor expression patterns on both monocytes and macrophages.^6–8^ Our focus has been on a single chromosomal locus incorporating the receptors CCR1, CCR2, CCR3, and CCR5.^9,10^ Of these receptors, CCR1, CCR2, and CCR5 (henceforth inflammatory chemokine receptors [iCCRs]) are clearly implicated in monocyte and macrophage function at inflamed sites. Using novel mouse models, we have demonstrated that, in 98% of inflammatory monocytes, CCR2 is the only iCCR that is significantly expressed.^9–11^ Mice bearing a compound deletion of the iCCR locus show the anticipated profound monocytopenia previously reported in CCR2 deficient mice confirming an essential, and nonredundant, role for CCR2 in monocyte egress from the bone marrow.^9^ Our data also demonstrate that CCR2 is central to recruitment of monocytes from the circulation to the tissue. Thus, the issue of redundancy of iCCR expression is not apparent in the context of monocytes, which are fully reliant on CCR2. In contrast to CCR2, expression of CCR1 and CCR5 becomes apparent once monocytes start to differentiate toward macrophages within peripheral tissue,^10^ and here things become more complex. To study iCCR biology, we previously generated compound transgenic reporter (REP) mice, with a recombineered bacterial artificial chromosome inserted into the genome, in which the coding sequence of each of the iCCR genes was replaced with sequences encoding spectrally distinct fluorescent proteins. Therefore, in REP mice, as an iCCR gene is expressed, expression of a corresponding fluorescent protein will also occur. Using these REP mice, we have shown that individual macrophages, within both resting and inflamed tissues, can express all combinations of CCR1, CCR2, and CCR5.^10^

The purpose of the present study was to examine the dynamics of iCCR expression by macrophages, to examine how dynamics alter as macrophages differentiate from monocytes, and to determine whether macrophage subsets display chaotic or specific patterns of receptor expression. In addition, we aimed to determine if iCCRs have a redundant or nonredundant role in the macrophage response to varied inflammatory stimuli. Here, we have used transcriptomic approaches to demonstrate that resting lung interstitial macrophages (IMs) expressing different combinations of iCCRs are transcriptionally distinct. Furthermore, we demonstrate, using single-cell RNA sequencing (scRNA-seq), that monocyte-to-macrophage differentiation is characterized by transition through an early monocyte-derived IM population, the largest proportion of which express CCR1, CCR2, and CCR5. We propose that this early recruited macrophage population, which we have termed *Retnla+* macrophages, express a combination of iCCRs to allow them to respond to a range of environmental cues. As macrophages terminally differentiate, they tailor iCCR expression along divergent trajectories, depending on the phenotype of terminally differentiated cell, thereby altering iCCR expression to align with macrophage function. Our data shed new light on the nature of monocyte-to-macrophage differentiation and demonstrate that iCCR expression on macrophages is not redundant or chaotic, but rather specific and malleable in response to discrete inflammatory agents.

## 2. Methods

### 2.1. Animals

CCR2 knockout (KO) (The Jackson Laboratory) (C57BL/6J background) and REP^10^ (C57BL/6N background) mice were housed in a specific pathogen–free animal facility at the University of Glasgow. Wild-type (WT) control mice were matched to the appropriate background of the transgenic mice. All mice were between 7 and 12 wk old, using a mixture of male and female mice for most experiments. Only female mice were used for scRNA-seq, as samples were combined into a single pool. Procedures were approved by the local University of Glasgow ethics committee and animal experimentation was carried out under a UK Home Office License in accordance with the revised Animal (Scientific Procedures) Act 1986.

### 2.2. Processing lung tissue

Lungs were perfused with 20 mL phosphate-buffered saline (PBS) and digested in Hanks’ Balanced Salt Solution (Thermo Fisher Scientific; 14170088) containing 0.44 Wunsch units of Liberase (Merck; LIBTM-RO). Samples were incubated on a thermoshaker running at 37 °C and 1,000 rpm, for 1 h. Digested samples were passed through a 70 µm filter, and the filter was flushed with PBS, to obtain a single-cell suspension. Cells were washed 2× in PBS supplemented with 2 µM EDTA.

### 2.3. Bulk RNA sequencing

**Figure 1:**
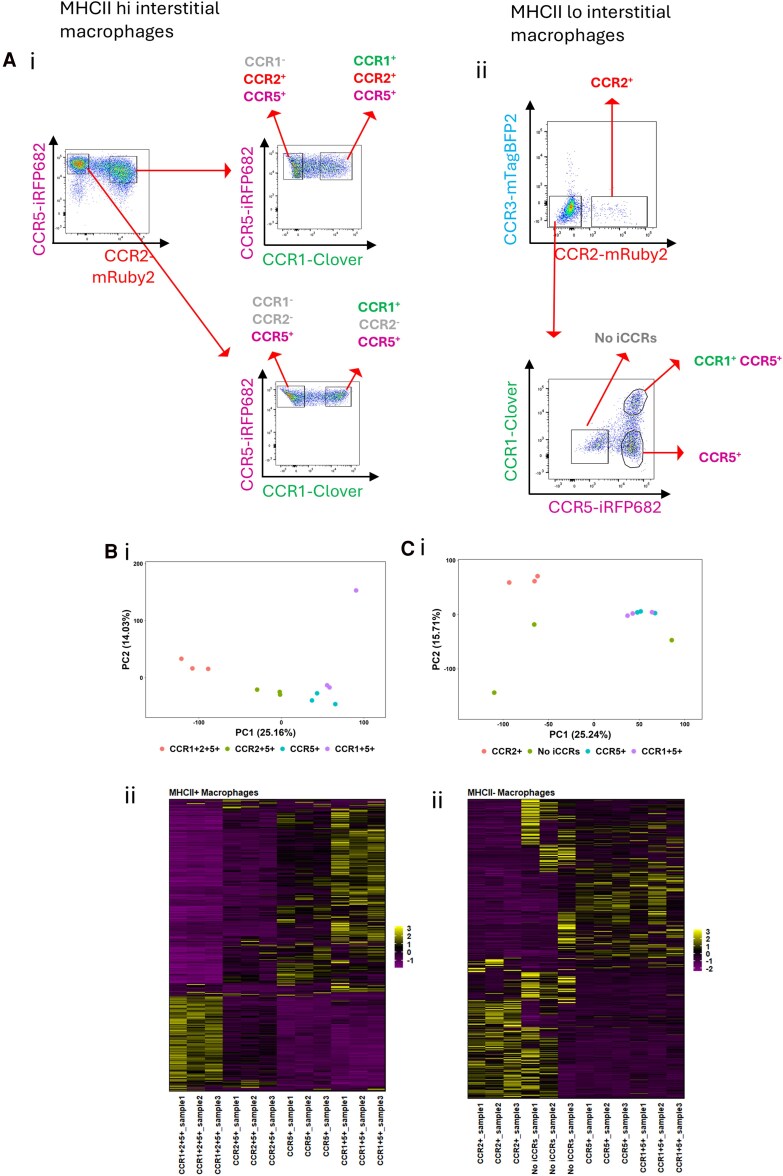
Bulk RNA-seq demonstrates that lung IM populations expressing different iCCR combinations are transcriptionally distinct. (A) (i) Flow cytometry plot showing the iCCR expression patterns of MHCII hi macrophages. (ii) Flow cytometry plot showing the iCCR expression patterns of MHCII lo macrophages. (B) (i) PC analysis scatter plot showing PC1 versus PC2 for MHCII hi macrophages expressing various combinations of iCCRs. Percentage variance is displayed in brackets for each PC. (ii) Heatmap showing significantly differentially expressed genes between MHCII hi macrophages displaying differing iCCR expression patterns. (C) (i) PC analysis scatter plot showing PC1 versus PC2 for MHCII lo macrophages expressing various combinations of iCCRs. Percentage variance is displayed in brackets for each PC. (ii) Heatmap showing significantly differentially expressed genes between MHCII lo macrophages displaying differing iCCR expression patterns. For heatmaps in panels Bii and Cii, significantly differentially expressed genes are displayed on the y-axis and were hierarchically clustered using Spearman distances with UPMGA agglomeration and mean reordering. Expression levels were row-scaled into z scores. Color represents expression level, with purple representing low expression and yellow representing high expression.

Cells were processed from the lung as described in Processing Lung Tissue. Samples were then incubated for 20 min at 4 °C with eFluor 506 viability stain (Thermo Fisher Scientific; 65-0866-14), diluted 1:1,000 in PBS. Fc receptor (FcR) binding was blocked by incubating the cells in FcR blocking reagent (Miltenyi Biotec, 130-092-575) Staining was performed using the following antibodies: anti-mouse CD45.2 PerCP-Cy5.5 (BioLegend; clone 104; cat. no 109828), anti-mouse/human CD11b APC-Cy7 (BioLegend; clone M1/70; cat no. 101226), anti-mouse CD64 Brilliant Violet 786 (BD Biosciences; clone: X54-5/7.1; cat. no. 569507), anti-mouse MHCII Brilliant Violet 605 (BioLegend; clone: M5/114.15.2; cat. no. 107639), anti-mouse F4/80 PE-Cy7 (Thermo Fisher Scientific; clone: BM8; cat no. 25-4801-82), anti-mouse Ly6C Alexa Fluor 700 (BioLegend; clone: HK1.4; cat. no. 128024), anti-mouse Ly6G Alexa Fluor 700 (BioLegend; clone: 1A8; cat no. 127622), and anti-mouse SiglecF Alexa Fluor 700 (BioLegend; clone: S17007L; cat. no. 155534). MHCII hi and MHCII lo macrophages expressing various combinations of iCCRs were flow cytometrically sorted on the BD FACSAria using the gating strategies presented in Figures 1A and S1A. Cells were collected into RLT buffer (Qiagen; 79216) containing 10 μL/mL of 2-Me and stored at −80 °C for RNA extraction. RNA extraction, library preparation, and bulk RNA sequencing (RNA-seq) was carried out as previously described.^11^ Briefly, RNA was extracted using an RNeasy Micro Kit (Qiagen) as per the manufacturer's instructions and messenger RNA libraries were prepared using the NEBNext Single Cell/Low Input RNA Library Prep Kit for Illumina (New England Biolabs; cat. no. E6420L). Samples were sequenced using paired-end sequencing on the NextSeq2000 sequencing platform (Illumina) (40 million reads sequencing depth). FastQ files were assessed using FastP,^12^ and aligned to the mouse reference genome (GRCm38.91) using STAR (2.7.10a).^13^ The expression and differential expression values were generated using DESeq2 (version 1.24)^14^ or differential comparisons, and an A versus B model with no additional covariates was used. All other parameters were left to default. The processed data were then visualized using Searchlight,^15^ specifying one differential expression workflow for each comparison, an absolute log2 fold cutoff of 1, and adjusted *P* value of 0.05.

### 2.4. Sample processing for scRNA-seq

Lungs from 3× female REP mice were perfused with 20 mL PBS and digested with Liberase, as described in Processing Lung Tissue. Digested samples were passed through a 70 µm filter to obtain a single-cell suspension and pooled together for sorting by FACS. Cells were stained first with eFluor 506 viability stain (65-0866-14), then with the following antibodies: anti-mouse/human CD11b PE-Cy7 (BioLegend; clone M1/70; cat no. 101216), anti-mouse Ly6G Brilliant Violet 711 (BioLegend; clone 1A8; cat no. 127643), and anti-mouse SiglecF Brilliant Violet 711 (BD Biosciences; clone E50-2440; cat no. 740764). CD11b+ Ly6G− SiglecF− cells were flow cytometrically sorted from total viable cells using the BD FACSAria and collected into 1% bovine serum albumin in PBS. Four more samples were flow cytometrically sorted from the CD11b+ Ly6G− SiglecF− gate based on iCCR expression (CCR1− CCR5+, CCR1+ CCR5+, CCR1+ CCR5−, CCR1− CCR5−); samples sorted based on iCCR expression were hashtagged using Total-SeqA antibodies (BioLegend; all antibodies were a combination of clones M1/42 and 30-F11 conjugated to different hashtag oligo sequences, cat. nos. 155801, 155803, 155805, and 155811) and pooled prior to library generation. A separate library was generated for the CD11b+ Ly6G− SiglecF− sample.

### 2.5. scRNA-seq and analysis

Single-cell libraries were generated and sequenced as previously described.^11^ Reads were aligned using the count function in 10x Genomics Cell Ranger, and outputs were imported into R (version 4.3.1; R Foundation for Statistical Computing) for downstream analysis using Seurat version 4.4.0.^16–20^ Single-cell hashtagged data were demultiplexed using the package CellhashR.^21^ The data were quality controlled by adjusting the range of features and counts included in downstream analysis. The range of features included in downstream analysis was 100 to 4,000, and the range of counts was 100 to 25,000. In addition, cells were removed if they did not meet the threshold for mitochondrial percentage (<5%). Data were normalized using both log normalization and SC-Transform. Integration features were identified using default features of the Seurat function SelectIntegrationFeatures. The function PrepSCTIntegration was then run on the SCT assay using the selected integration features. Thirty principal components (PCs) were included in the function FindIntegrationAnchors to identify the anchors required for integration. Thirty PCs were selected based on the results of an Elbow Plot displaying PC1 to PC50 (data not shown). Finally, data were integrated using the Seurat function IntegrateData, again including 30 PCs. Seurat was updated to version 5 to run downstream analysis. Data were plotted and clustered using the functions FindVariableFeatures, ScaleData, RunPCA (npcs = 10), RunUMAP (reduction = “pca”, dims = 1:10), and FindNeighbours (reduction = “pca”, dims = 1:10 and FindClusters (resolution = 0.5). The effect of cell cycle was assessed using the function CellCycleScoring and the cell cycle score was regressed from the data for downstream analysis. Uniform Manifold Approximation and Projection (UMAP) dimensionality reduction was applied for visualization of the data in Dimensional Reduction Plots and Feature Plots. Pseudotime analysis was performed in R (version 4.3.1) using Monocle3.^22,23^ Briefly, the integrated Seurat object was transformed into a Cell Data Set (CDS) object using the default parameters of the function as.cell_data_set. Preprocessing of the CDS object was then carried out using the function preprocess_cds (num_dim = 100). Seurat clusters from previous analysis were applied to the CDS object. Pseudotime analysis was then performed using the functions:learn_graph (use_partitions = TRUE), order_cells (reduction method = UMAP), and estimate_size_factors. The default statistical tests were used for all Seurat and Monocle3 functions.

### 2.6. Flow cytometry

#### 2.6.1. Lung macrophages

Mice were culled via CO_2_ inhalation and lungs perfused with 20 mL PBS. Lungs were processed into a single cell suspension as described in Processing Lung Tissue. Samples were then stained with eFluor 506 viability stain, diluted 1:1,000 in PBS. FcR binding was blocked by incubating the cells in FcR blocking reagent (Miltenyi Biotec; cat. no. 130-092-575). For experiments with CCR2 KO mice and equivalent WT control mice, staining was performed using the following antibodies: anti-mouse CD45.2–APC-Fire 750 (BioLegend; clone: 104; cat. no. 109852), anti-mouse Ly6G–Brilliant Violet 711 (BioLegend; clone: 1A8; cat. no. 127643), anti-mouse Ly6C–Alexa Fluor 488 (BioLegend, clone: HK1.4; cat. no. 128022), SiglecF–Brilliant Ultra-violet 737 (BD Biosciences; clone: E50-2440; 570753), F4/80–PE (BioLegend; clone: W20065B; cat. no. 111604), anti-mouse CD11b–PE-Cy7 (BioLegend; clone M1/70), anti-mouse CD64–Brilliant Violet 786 (BD Biosciences; clone: X54-5/7.1; cat. no. 569507), anti-mouse MHCII Brilliant Violet 605 (BioLegend; clone: M5/114.15.2; cat. no. 107639), CD11c–Alexa Fluor 700 (BD Biosciences; clone: HL3; cat. no. 560583), anti-mouse CD2–APC (BioLegend; clone: RM2-5; cat. no. 100112), and anti-mouse CD226–PerCP-Cy5.5 (BioLegend; clone: 10E5; cat. no. 128814). For experiments using REP mice, antibodies targeting Ly6G, MHCII, and CD226 were the same as stated previously for the CCR2 KO experiment. Additional antibodies used were anti-mouse CD45 APC-Cy7 (BioLegend, clone: 30-F11; cat. no. 103116), anti-mouse/human CD11b Brilliant Ultra Violet 805 (BD Biosciences; clone: M1/70; cat. no. 568345), anti-mouse SiglecF Brilliant Violet 711 (BD Biosciences; clone: E50-2440; cat no. 740764), anti-mouse F4/80 Brilliant Violet 650 (BioLegend; clone: BM8; cat. no. 123149), and anti-mouse CD2 PE-Cy7 (BioLegend; clone RM2-5; cat. no. 100113). Antibody staining was performed at 4 °C for 20 min. Samples were acquired using BD LSRFortessa (BD Biosciences) and analyzed using FlowJo (v10; BD Biosciences).

#### 2.6.2. Bone marrow–derived macrophages

Bone marrow–derived macrophages (BMDMs) were cultured and treated as described subsequently, then lifted from plastic using Trypsin-EDTA (0.5%) (Thermo Fisher Scientific; 25300054) and stained with viability stain eFluor 506 diluted 1:1,000 in PBS. FcR binding was blocked by incubating the cells in FcR blocking reagent and staining was performed by incubating cells with the following cocktail of antibodies for 20 min at 4 °C: CD11b–APC-Cy7 (BioLegend; clone = M1/70) and F4/80–Brilliant Violet 785 (BioLegend; clone = BM8). Samples were acquired using BD LSRFortessa and analyzed using FlowJo (v10).

### 2.7. Culture of BMDMs and treatment with inflammatory mediators

BMDMs were cultured as previously described.^24^ Briefly, tibias and femurs were collected from culled mice and BM was flushed out and strained through a 70 μm cell strainer, and red cells lysed using ACK lysis buffer (Gibco; A1049201), to obtain a single-cell suspension of leukocytes. Cells suspended in Glasgow's Minimal Essential Medium (Gibco; 11710035 supplemented with 15% L929 conditioned media, 10% fetal bovine serum (Thermo Fisher Scientific; 10500064), 1% L-glutamine (Thermo Fisher Scientific; 25030024), 100 mM sodium pyruvate solution (Sigma-Aldrich; S8636), 1% MEM nonessential amino acids (Gibco; 1140-035), 50 μM b-ME (Gibco; 31350-010), and 0.2% Primocin (InvivoGen; ant-pm-1) were seeded at 1.2 × 10^6^ cells per well of a 6-well plate. Cells were cultured for 7 d, to allow macrophages to differentiate, and treated with Toll-like receptor (TLR) agonists for the final 24 h. TLR agonists used were Pam3CSK4 (TLR1/2 agonist used at 150 ng/mL; InvivoGen; tlrl-pms), Poly(I:C) (HMW) (TLR3 agonist used at 1 µg/mL; InvivoGen; tlrl-hklm), Poly(I:C) (LMW) (TLR3 agonist used at 5 µg/mL; InvivoGen; tlrl-picw), LPS-O127:B8 (TLR4 agonist used at 100 ng/mL; Sigma Aldrich; L45-16), LPS-EK (TLR4 agonist used at 5 µg/mL; InvivoGen; tlrl-eklps), FLA-ST (TLR5 agonist used at 5 µg/mL; InvivoGen; tlrl-stfla), and FSL-1 (TLR2/6 agonist used at 50 ng/mL; InvivoGen; tlrl-fsl).

### 2.8. Intranasal inflammatory mediator treatments

Mice were anesthetized using inhaled isoflurane (4% v/v + 2 L O_2_/min) and treated intranasally with 25 µL lipopolysaccharide (LPS) (1,000 µg/mL) (Invitrogen; 00-4976-03), 25 µL Poly(I:C) (1,000 ug/mL) (InvivoGen; tlrl-pic), or 25 µL PBS as a control. Four hours later, mice were culled via intraperitoneal injection of the 200 µg/mL pentobarbital sodium solution, Dolethal (vetoquinol). Lungs were perfused with 20 mL PBS and collected into RNAlater Stabilization Solution (Thermo Fisher Scientific; AM7020) for downstream processing.

### 2.9. Gene expression analysis using TaqMan Array Cards

Tissue samples intended for RNA extraction were stored in RNAlater for at least 24 h. Tissues were lysed and whole RNA was extracted using TRIzol Reagent (Thermo Fisher Scientific; cat. no. 15596026) chloroform extraction, followed by column cleanup using PureLink RNA Mini Kit (Invitrogen; cat. no. 4387406) with an on-column DNase (Qiagen; 79254) digest. RNA was converted to complementary DNA (cDNA) using the High-Capacity RNA-to-cDNA Kit (Applied Biosystems; cat. no. 10704217). The expression of 48 genes, including housekeeping genes, was determined using custom made TaqMan Array Cards (Thermo Fisher Scientific; cat. no. 4342253, design ID RTRWE3D). A total of 1 µg of cDNA with TaqMan Fast Advanced Master Mix (Thermo Fisher Scientific; cat. no. 4444557) was loaded per slot and array cards were run on a QuantStudio 7 Flex (Thermo Fisher Scientific; cat. no. 4485701) real-time PCR system. ^ΔΔ^CT values of target genes were calculated toward 18s RNA.

### 2.10. Statistics

Statistics used in bulk RNA-seq and scRNA-seq analysis performed in R were previously described in their respective sections. All other statistical tests were carried out using GraphPad Prism 10 software (GraphPad Software). Data were tested for normality using a Shapiro-Wilk test. Normally distributed data were analyzed by 1-way analysis of variance and nonparametric data were analyzed by Kruskal-Wallis test.

## 3. Results

### 3.1. Bulk RNA-seq demonstrates that IMs expressing different combinations of iCCRs are transcriptionally distinct

Bulk RNA-seq was performed on cells sorted by FACS from MHCII hi and MHCII lo IM populations based on iCCR expression patterns, in order to determine if cells expressing different combinations of iCCRs are transcriptionally distinct and thus possess discrete functionality. For this analysis, different iCCR-expressing macrophage populations were flow cytometrically sorted from REP mice. The gating strategies are presented in Figures 1A and  S1A. Macrophages were gated first on MHCII expression, to delineate MHCII hi versus MHCII lo macrophages, then gated on iCCR expression patterns. iCCR populations FACS sorted from the MHCII hi gate included CCR5+ cells, CCR1+ CCR5+ cells, CCR2+ CCR5+ cells, and CCR1+ CCR2+ CCR5+ cells (Fig. 1Ai). MHCII lo IMs were also FACS sorted based on iCCR expression patterns. Cells FACS sorted from the MHCII lo gate included iCCR− cells, CCR2+ cells, CCR5+ cells, and CCR1+ CCR5+ cells (Fig. 1Aii). Cells from each of these gates were collected for RNA-seq. PC analysis of the MHCII hi IMs revealed that each population clustered separately on a PC1 vs PC2 scatterplot, demonstrating that there are transcriptional differences between each of the iCCR expressing populations (Fig. 1Bi). This is further demonstrated by the heatmap in Figure 1Bii, which shows significantly differentially expressed genes between MHCII hi IMs expressing different iCCR combinations. This analysis suggests that the CCR1+ CCR2+ CCR5+ population has the most highly distinct gene expression profile of all MHCII hi IMs, but that all populations are unique (Fig. 1Bii).

Analysis of MHCII lo IMs revealed that CCR2 expression was depleted compared with MHCII hi IMs (Fig. 1Aii). In addition, MHCII lo IMs also contained a population negative for iCCR expression (Fig. 1Aii). PC analysis of MHCII lo IMs revealed that, while iCCR− cells and CCR2+ cells formed distinct clusters on a PC1 vs PC2 scatterplot, CCR1+ CCR5+ and CCR5+ cells clustered together (Fig. 1Ci). A heatmap depicting significantly differentially expressed genes between MHCII lo IM populations confirms this observation (Fig. 1Cii), demonstrating CCR2+ and iCCR+ cells to have unique gene expression patterns, whereas the gene expression patterns for CCR1+ and CCR1+ CCR5+ MHCII lo IMs was highly similar.

Essentially, the bulk RNA-seq data show that MHCII hi and MHCII lo IMs expressing various combinations of iCCRs are transcriptionally distinct (with the exception of CCR1+ CCR5+ and CCR5+ MHCII lo IMs), suggesting discreet roles for iCCRs in the inflammatory response.

### 3.2. scRNA-seq confirms that differential iCCR expression patterns mark distinct IM subsets

Next, scRNA-seq was employed to investigate the transcriptional relationship between different iCCR-expressing IM populations in the resting lung. IMs were enriched from the lungs of REP mice^10^ by FACS sorting CD11b+ SiglecF− Ly6G− cells, in order to deplete alveolar macrophages, eosinophils, and neutrophils from the analysis. The enriched population was then FACS sorted based on the expression of CCR1 and CCR5 reporters (the gating strategy for sorts is demonstrated in Fig. S1B). The resulting 4 populations of enriched cells (CCR1− CCR5+, CCR1+ CCR5+, CCR1+ CCR5−, CCR1− CCR5−) were separately hashtagged using different TotalSeq-A antibodies in order to accurately identify the iCCR-expressing populations during sequencing analysis. This step was important, as transcript and protein expression levels do not always align for CCR1 and CCR5, making transcript levels unreliable as an indicator of receptor protein expression.^25,26^ Hashtagged samples were integrated together with an enriched, nonhashtagged sample of CD11b+ SiglecF− Ly6G− lung cells.

**Figure 2:**
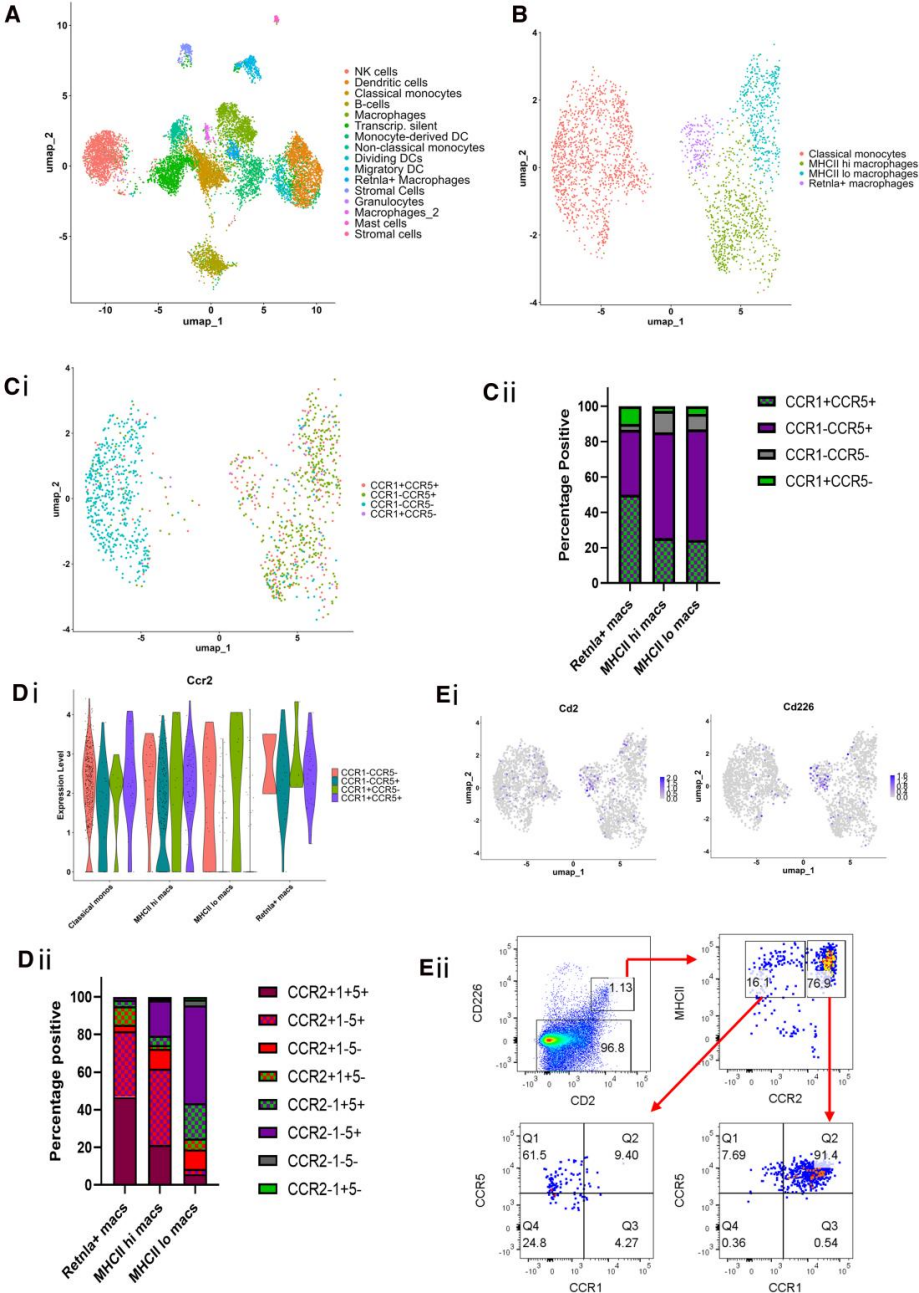
Single-cell scRNA-seq reveals that subsets of lung IMs have unique iCCR expression patterns and that iCCR expression is not random. (A) UMAP visualization of CD11b+ Ly6G− SiglecF− lung cells. (B) UMAP plot of data subsetted from panel A depicting cells positive for monocyte or macrophage markers. Nonclassical monocytes were also removed from the dataset. Colors represent unique clusters termed “classical monocytes,” “*Retnla+* macrophages,” “MHCII hi macrophages,” and “MHCII lo macrophages.” (C) (i) UMAP displaying hashtagged populations and how they correspond to the monocyte and macrophage clusters presented in panel B. Blue = CCR1− CCR5−; red = CCR1+ CCR5+; green = CCR1− CCR5+; purple = CCR1+ CCR5−. UMAP plots in panels B and C have the same coordinates. (ii) Bar graph presenting the proportion of hashtagged cells corresponding to each macrophage population. (D) (i) Violin plot showing the relative expression of *Ccr2* by hashtagged populations across the 4 clusters presented in panel B. (ii) Bar graph presenting combinatorial iCCR expression across the 3 macrophage populations presented in panel B. Cells were considered positive for *Ccr2* if relative expression was >1. Feature plots depicting the expression of *Cd2* and *Cd226* within the scRNA-seq dataset. UMAP coordinates are identical to the plot shown in panel B. (i) Antibodies against CD2 and CD226 were used to identify *Retnla+* macrophages by flow cytometry and assess MHCII and iCCR expression in the CD2+ CD226+ population. DC, dendritic cell; NK, natural killer.

Bioinformatic analysis revealed 16 clusters of cells, including natural killer cells, dendritic cells, monocytes, macrophages, B cells, granulocytes, and stromal cells (Fig. 2A). A full list of genes differentially expressed between clusters can be found in Table S1. The data were then subsetted to depict only monocytes and macrophages. Monocytes clearly separated into 2 distinct populations that could be delineated by Ly6C expression, the largest population consisting of Ly6C hi classical monocytes, with a smaller population depicting Ly6C lo nonclassical monocytes. In the current study, no trajectory could be identified between nonclassical monocytes and IMs (Fig. S3Ai, Aii) in the resting lung; therefore, nonclassical monocytes were removed from downstream analysis and the data rescaled (Fig. 2B). A full list of genes differentially expressed between the monocyte and macrophage clusters depicted in Figure 2B can be found in Table S2.

Similar to previous studies,^27^ we found that lung IMs can be separated into 3 distinct clusters based on gene expression patterns (Fig. 2B). The top 20 differentially expressed genes between these 3 clusters is depicted in the heatmap in Figure S1C, demonstrating that gene expression between these 3 clusters was highly distinct. A distinguishing feature of the smallest IM cluster was high *Retnla*^28^ gene expression; therefore, this cluster was termed *Retnla+* macrophages. The remaining macrophage clusters were identified as MHCII hi and MHCII lo IMs. After clustering the cells, hashtagged cells were subsetted from the data, keeping the same UMAP coordinates. The purpose of this was to observe where each hashtagged (i.e. CCR1 and/or CCR5 expressing) population fell within the UMAP and, therefore, what macrophage population they corresponded to (Fig. 2Ci). In agreement with previous work,^11^ classical monocytes were almost entirely negative for CCR1 and CCR5 expression. The largest hashtagged population mapping to the *Retnla+* macrophage cluster was the CCR1+ CCR5+ population. Interestingly, CCR1 and CCR5 expression was largely uniform across MHCII hi and MHCII lo macrophages, with the majority of cells in both clusters being CCR1− CCR5+ (Fig. 2Ci). Figure 2Cii presents a bar graph depicting the percentage of hashtagged populations mapping to each cluster. This demonstrates that 86.6% of hashtagged *Retnla+* macrophages were positive for CCR5. Similar proportions of CCR5+ cells were observed in the hashtagged MHCII hi macrophage cluster (85.2% CCR5+) and hashtagged MHCII lo macrophage cluster (86.9% CCR5+). In contrast, 60% of *Retnla+* macrophages were positive for CCR1, whereas only 30% of MHCII hi macrophages and 32% of MHCII lo macrophages were positive for CCR1. Thus, CCR1 and CCR5 expression distributes nonrandomly across the 3 identified macrophage clusters.

We next examined the effect of layering *Ccr2* expression onto the hashtagged populations. Hashtagging was not required for CCR2 as reporter protein levels correlate well with transcript levels.^10^ We looked at *Ccr2* gene expression across the hashtagged populations depicted in Figure 2Ci. A cell was considered positive for *Ccr2* if relative expression was >1. As expected, we observed high *Ccr2* expression in the CCR1− CCR5− hashtagged population, which primarily mapped to classical monocytes (Fig. 2Di). A minority (11.7%) of macrophages were also CCR1− CCR5−, and of these, 85% expressed *Ccr2*. CCR2+ CCR1− CCR5− cells comprised 33% of *Retnla+* macrophages, 10.7% of MHCII hi IMs, and 10.4% of MHCII lo IMs, respectively. In the CCR1− CCR5+ hashtagged population, *Ccr2* was expressed in *Retnla+* macrophages and MHCII hi IMs but was largely absent from MHCII lo IMs (Fig. 2Di). CCR2+ CCR1− CCR5+ cells made up 35% of *Retnla+* macrophages and 40.4% of MHCII hi IMs but only 2.7% of MHCII lo macrophages (Fig. 2Di). CCR1+ CCR5− cells were rare and made up only 2.8% of total hashtagged cells. CCR2+ CCR1+ CCR5− cells mapped primarily to *Retnla+* macrophages, making up 10% of the *Retnla+* macrophage population (Fig. 2Di). Similar to the CCR1− CCR5+ population, *Ccr2* was expressed in CCR1+ CCR5+ cells mapping to *Retnla+* macrophages and MHCII hi IMs but was absent from MHCII lo IMs. *Retnla+* macrophages had the highest proportion of CCR2+ CCR1+ CCR5+ cells (46.7%), while 21.3% of MHCII hi IMs and 5.8% of MHCII lo IMs were CCR2+ CCR1+ CCR5+. Looking at the gene expression level, we found that the log2 fold change of *Ccr2* was 0.611 in classical monocytes (*P* value = 1.03 × 10^−20^) and 0.579 in *Retnla+* macrophages (*P* value = 4.72 × 10^−6^), compared with all other clusters. *Ccr2* was not a significant marker gene in MHCII hi or MHCII lo IMs (Table S2). The combinatorial expression of *Ccr2*, CCR1, and CCR5 by the 3 observed macrophage populations is summarized in Figure 2Dii.

Due to *Retnla+* macrophages having the highest proportion of CCR1+ CCR2+ CCR5+, we hypothesized that they, in fact, correlate to the CCR1+ CCR2+ CCR5+ MHCII hi macrophage population described in the bulk RNA-seq (Fig. 1), potentially explaining why the gene expression pattern of CCR1+ CCR2+ CCR5+ MHCII hi IMs were highly distinct (Fig. 1Bi, Bii). To test this, we used the function FindAllMarkers in Seurat to identify genes expressing cell surface molecules that could be used to identify *Retnla*+ macrophages by flow cytometry. This was necessary because RELMα, encoded by *Retnla*, is not a cell surface molecule. CD2 and CD226 were selected as alternative markers of *Retnla*+ macrophages for flow cytometry analysis. The specific expression of the genes encoding CD2 (*Cd2*) and CD226 (*Cd226*) in the single-cell sequencing data is presented in the feature plots shown in Figure 2Ei, demonstrating that expression is highly specific to *Retnla+* macrophages. Lung IMs were stained for flow cytometry analysis (gating strategy provided in Fig. 2Eii and  Fig. S2A), which confirmed that CD2+ CD226+ macrophages (i.e. *Retnla+* macrophages) had high MHCII expression and were primarily CCR1+ CCR2+ CCR5+ (Fig. 2Eii). Figure S2B shows expression of key *Retnla+* macrophage marker genes identified by scRNA-seq, by the macrophage populations analyzed by bulk RNA-seq (Fig. 1). The marker genes were selected first by using the FindAllMarkers function in Seurat. Significant genes correlating to *Retnla+* macrophages were then sorted based on the difference between pct.1 and pct.2. The top 30 of these genes was then sorted by highest to lowest log2 fold change. Using this method, the top 14 *Retnla+* macrophage marker genes, identified from the single-cell sequencing dataset, are depicted in Figure S2B, with the addition of *Ccr2*. Importantly, these data demonstrate that the majority of these *Retnla+* macrophage marker genes are preferentially expressed by MHCII hi CCR1+ CCR2+ CCR5+ macrophages analyzed by bulk RNA-seq, confirming the transcriptional relatedness of these populations.

To summarize, the CCR1+ CCR2+ CCR5+ MHCII hi IM population described in the bulk RNA-seq analysis (Fig. 1) are a distinct population of lung IMs that highly express *Retnla* and likely have a unique functional role in the lung. MHCII hi and MHCII lo IMs differ in their iCCR expression patterns; MHCII hi IMs are predominantly CCR1− CCR2+ CCR5+, whereas MHCII lo IMs are predominantly CCR1− CCR2− CCR5+, and express very little *Ccr2* compared with MHCII hi IMs, confirming the bulk RNA-seq results. Together, the bulk RNA-seq and scRNA-seq confirm that iCCR expression is specifically patterned across macrophage subsets in the lung and is not random or stochastic.

### 3.3. Pseudotime analysis reveals that IMs develop along two distinct lineages

**Figure 3:**
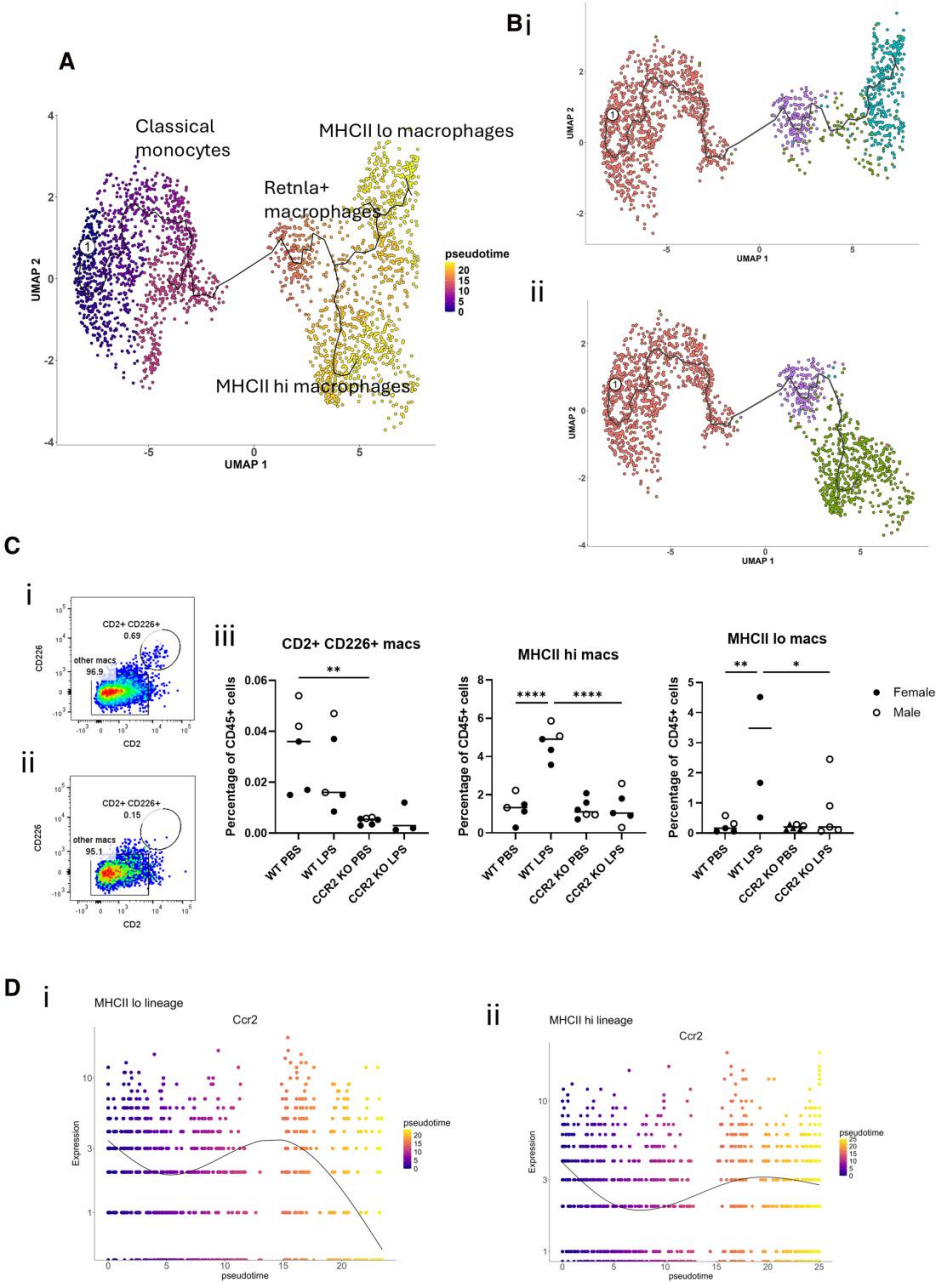
Pseudotime analysis of lung monocyte and macrophage populations demonstrates two distinct macrophage lineages. (A) UMAP plot demonstrating the trajectory from monocytes to macrophages. Colors represent the stage of pseudotime: purple indicates early in the trajectory and yellow indicates late in the trajectory. The clusters identified in Figure 2B are labeled. (B) (i) UMAP showing the MHCII lo macrophage lineage trajectory. (ii) UMAP showing the MHCII hi macrophage lineage trajectory. (C) (i) Representative flow cytometry plot showing CD2+ CD226+ expression on F4/80+ macrophages in WT mice. (ii) Representative flow cytometry plot showing CD2+ CD226+ expression of F4/80+ macrophages in CCR2 KO mice. (iii) Quantification of CD2+ CD226+ macrophages (representing *Retnla+* macrophages), MHCII hi macrophages, and MHCII lo macrophages in WT and CCR2 KO mice at rest and during LPS induced inflammation. Data are expressed as percentage of CD45+ cells. Symbol shape represents sex: filled circles indicate female mice and hollow circles indicate male mice. Data were normally distributed and analyzed by 1-way analysis of variance. n = 5 or 6. *P < 0.5, **P < 0.01, ****P < 0.0001. (D) (i) *Ccr2* expression across pseudotime in the MHCII lo lineage. (ii) *Ccr2* gene expression across pseudotime in the MHCII hi lineage. The color scale represents the stage of pseudotime: purple indicates early in the trajectory and yellow indicates late in the trajectory.

In order to understand if changes in IM iCCR expression patterns were altered along a trajectory or if they delineated unrelated macrophage populations, we performed pseudotime analysis using Monocle3.^22^ As it is widely reported that IMs are monocyte derived,^29,30^ classical monocytes were selected as the root of the trajectory. These results confirmed that the first macrophage population to arise from monocytes along this trajectory were *Retnla+* macrophages (Fig. 3A), the largest fraction of which are CCR2+ CCR1+ CCR5+. The trajectory then branched and created two major lineages that corresponded to MHCII lo IMs (Fig. 3Bi) and MHCII hi IMs (Fig. 3Bii). The top 5 differentially expressed genes across pseudotime were calculated for the MHCII lo (Fig. S3Bi) and MHCII hi trajectories (Fig. S3Bii). As cells differentiate into MHCII lo IMs, they upregulate genes associated with an M2 macrophage phenotype, including *Cfh*, *Fcna*, *Marco*, and *Mrc1*. In contrast, as cells differentiate into MHCII hi IMs they downregulate *Fn1*, *Samhd1*, and *Thbs1* and upregulate *Rgs1*, suggesting that these cells are digressing from an M2 phenotype to become more inflammatory.

Flow cytometry was employed to confirm that *Retnla+* macrophages do indeed derive from monocytes, as the pseudotime analysis suggests. To assess this, WT mice, and CCR2 KO mice (which display profound monocytopenia in the circulation), were treated intranasally with LPS for 24 h to induce inflammation in the lung and drive monocyte recruitment. In keeping with their monocytic origin, while *Retnla+* macrophages (CD2+ CD226+) were easily detectable in resting and inflamed WT lungs, they were almost entirely absent from resting and inflamed lungs of CCR2 KO mice (Fig. 3Ci, Cii). Interestingly, and in contrast to MHCII hi and lo macrophages, LPS did not increase *Retnla+* macrophage numbers in WT lungs, suggesting that differentiation into *Retnla+* macrophages is an “at rest” process and does not occur in response to inflammatory stimuli (Fig. 3Ciii). This is in keeping with the immunoregulatory role of RELMα in the lung that has previously been reported.^28,31,32^

Due to the changes observed in *Ccr2* expression between IM subsets, presented in Figure 2D, we measured *Ccr2* across pseudotime for the two observed lineages. As expected, we found that *Ccr2* expression peaked in *Retnla+* macrophages then dramatically decreased across pseudotime along the MHCII lo lineage (Fig. 3Di). Although *Ccr2* expression did decrease across pseudotime in the MHCII hi lineage (Fig. 3Dii), it was to a much lesser extent than the MHCII lo lineage. It is interesting to note that *Ccr2* expression is downregulated as monocytes differentiate into IMs and is then upregulated again by *Retnla+* macrophages early in the trajectory (Figs 3Di, Dii). This suggests that *Retnla+* macrophages, and MHCII hi macrophages, have a specific biological requirement for CCR2 in addition to CCR1 and CCR5.

Overall, these data demonstrate that the *Retnla+* macrophages expressing all 3 iCCRs sit on a trajectory intermediate between monocytes and terminally differentiated MHCII hi and MHCII lo IMs.

### 3.4. iCCR expression on macrophages can be patterned by inflammatory mediator exposure

**Figure 4:**
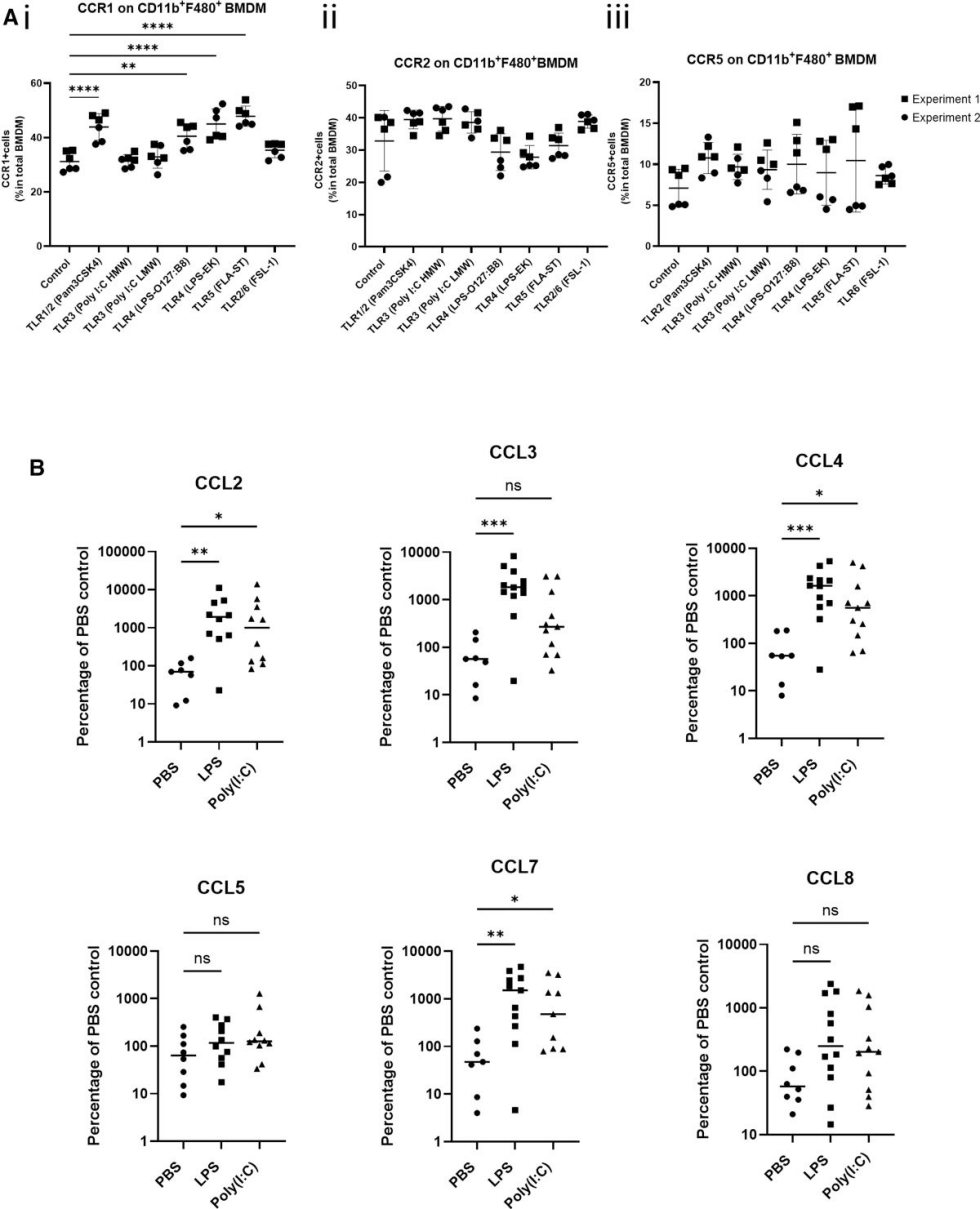
Inflammatory stimuli alter iCCR expression on BMDMand ligand expression in the lung. (A) iCCR expression on BMDMs in vitro after 24 h inflammatory mediator stimulation. BMDMs were first gated based on viability and CD11b and F4/80 expression (gating strategy provided in Fig. S4A) (i) Percentage of total F4/80+ BMDMs expressing CCR1 after 24 h TLR ligand stimulation. (ii) Percentage of total F4/80+ BMDMs expressing CCR2 after 24 h TLR ligand stimulation. (iii) Percentage of total F4/80+ BMDMs expressing CCR5 after 24 h TLR ligand stimulation. Data in panel A are pooled from 2 independent experiments denoted by symbol shape. Data were normally distributed and analyzed by 1-way analysis of variance. n = 6. **P < 0.01, ****P < 0.0001. (B) CCL gene expression in the lung 4 h post–intranasal treatment with TLR ligands, LPS, and Poly(I:C). CCLs were measured using TaqMan array cards. ^ΔΔ^CT values of target genes were calculated toward 18s RNA and then presented as percentage of PBS control. Data were non-normally distributed and analyzed by Kruskal-Wallis test, comparing each treatment condition with the PBS control. n = 7–10. *P < 0.05, **P < 0.01, ***P < 0.001. ns, not significant.

To test the hypothesis that the unique iCCR patterning observed on IM populations is related to discrete functionality of macrophages, indicating nonredundant iCCR expression, we generated BMDMs from REP mice and treated them for 24 h with a range of TLR ligands representative of a variety of pathogens and inflammatory contexts. Flow cytometric analysis (gating strategy in Fig. S4A) of treated cultures demonstrated that CCR1 expression was increased in response to TLR1/2, TLR4, and TLR5 ligands, but not TLR3 or TLR2/6 ligands, suggestive of regulation by bacterial, but not viral, pathogens (Fig. 4Ai). In contrast, CCR2 (Fig. 4Aii) and CCR5 (Fig. 4Aiii) expression was not significantly increased in response to any TLR ligand. The tailored response of CCR1 to TLR signaling in response to bacterial associated ligands suggests a nonredundant role for CCR1 expression on macrophages.

### 3.5. Inflammatory chemokine induction in inflamed lung is differentially regulated by bacterial and viral mimetics

To complement the receptor analyses, we next examined inflammatory chemokine expression in the lung in response to inhaled TLR ligands indicative of bacterial (LPS) and viral [Poly-(I:C)] pathogens. Lungs were harvested 4 h after TLR ligand treatment and chemokine transcript levels in lung tissue assessed using TaqMan Array cards. These studies focus on CCL2, CCL3, CCL4, CCL5, CCL7, and CCL8 expression, as these chemokines are known ligands of iCCRs. Expression of these chemokines in the inflamed lung relative to other CC ligands is shown in Figure S4B. As shown in Figure 4B, with the exception of CCL5 and CCL8, each of these ligands is robustly induced in response to LPS. Notably, while CCL2, CCL4, and CCL7 are also strongly induced in response to Poly(I:C), CCL3 is significantly increased only in response to LPS. As CCL3 is a ligand for CCR1, which, as shown in Figure 4A, is significantly upregulated on BMDMs in response to TLR ligands associated with bacterial infection, it is likely that CCL3 signaling via CCR1 has a preferential role in the macrophage response to bacterial infection.

**Figure 5:**
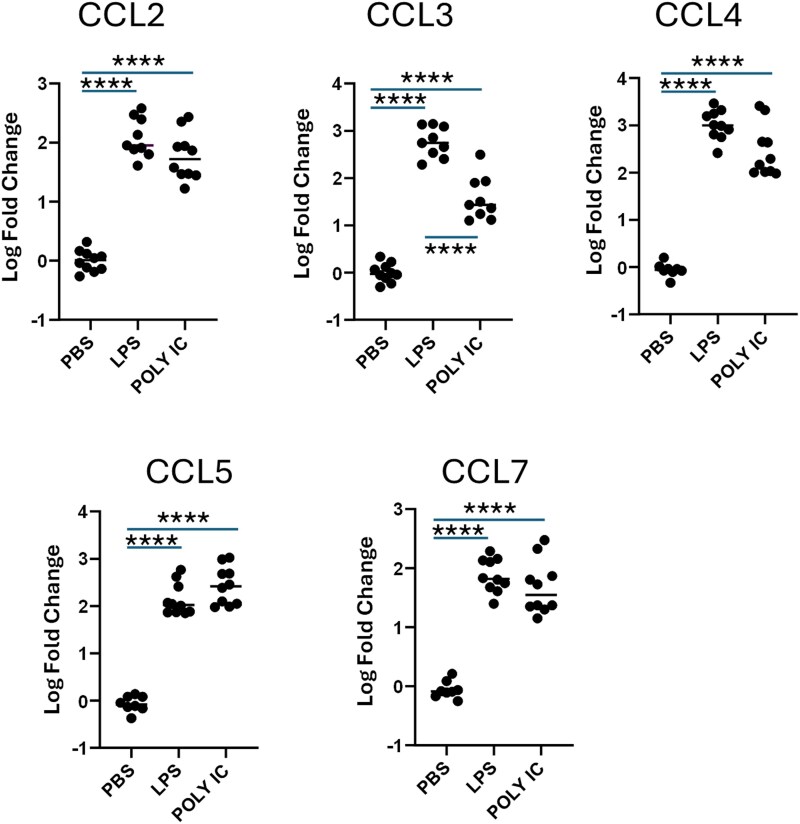
Expression of CCLs in the skin 4 h after intradermal injection with inflammatory mediators. CCL gene expression in mouse dorsal skin 4 h following intradermal injection with LPS or Poly(I:C). Data were normally distributed and analyzed by 1-way analysis of variance. n = 7–10. ****P < 0.0001.

To understand if CCL expression observed in response to LPS and Poly(I:C) was tissue specific, or representative of a more general response, we generated data examining chemokine regulation following intradermal injection of the same agents (Fig. 5). Expression of CCL2, CCL3, CCL4, and CCL7 was highly similar in the skin compared with the lung in response to both TLR ligands.

To summarize, increased expression of CCL3 is preferentially observed in response to bacterial but not viral agents after both pulmonary and cutaneous administration. The specific increase in CCL3 and CCR1 in response to bacterial ligands, supports the sequencing data presented in Figures 1 and 2, which suggested a nonredundant role for iCCRs in lung IM populations based on nonstochastic expression patterns across IM subsets and significant differential gene expression between IM subsets expressing different iCCR combinations.

## 4. Discussion

For many years, it has been argued that, particularly on inflammatory cells, chemokine receptors function in a redundant manner by providing “fall-back” options to protect the robustness of the inflammatory response.^6,7^ However, this notion has not been rigorously tested. Here, we have examined this focusing on a tight genomic locus incorporating 4 chemokine receptors that we refer to as iCCRs. These receptors have been reported to be expressed in multiple combinations on inflammatory cells and have therefore been suggested to exemplify the notion of redundancy. Using multichemokine receptor reporter mice,^10^ we have previously confirmed that macrophages can indeed display essentially all combinations of CCR1, CCR2, and CCR5 but determining the relevance of this for redundancy has been difficult. In part this is because clear roles for CCR1 and CCR5, in macrophage biology, have not yet been defined^9^ and thus determining potential functional redundancy is problematic. Here, we have used transcriptomic approaches to test the hypothesis that chemokine receptors do not function in a redundant manner and that macrophages expressing different iCCR combinations represent distinct macrophage populations.

Using both bulk RNA-seq and scRNA-seq, we have demonstrated that for both MHCII hi and MHCII lo IMs cells expressing different iCCR combinations are transcriptionally distinct. Furthermore, bioinformatic analysis of scRNA-seq data show that chemokine receptor expression can be used to define a trajectory from CCR2+ inflammatory monocytes, via a *Retnla*+ intermediate macrophage population coexpressing CCR1, CCR2, and CCR5, to mature macrophages expressing various combinations of CCR1, CCR2, and CCR5. Notably, CCR5 dominates receptor expression patterns in MHCII hi and lo macrophages frequently in combination with CCR2 in MHCII hi cells. Some expression of CCR1 is noted but it is limited. Overall, these data suggest that macrophage differentiation from inflammatory monocytes proceeds via a *Retnla*+ intermediate population expressing all 3 iCCRs and that expression is subsequently refined, and restricted, on fully mature macrophages. These data are from IMs derived from resting lungs and suggest that the predominant default in mature cells is CCR5 expression. Interestingly, our analysis of in vitro differentiated macrophages (Fig. 4Aii) indicates that CCR2 expression is unaffected by TLR ligand expression in these cells. Notably, previous reports indicate that CCR2 expression is rapidly downregulated in monocytes,^33^ and while we see no effect on macrophages, this is broadly in keeping with the data shown in Figure 3Di and Dii.

Expression of multiple cell surface proteins in precursor cells, with progressive restriction upon differentiation, is also seen in other situations^34,35^ suggesting that this may be a more general phenomenon in biology. Furthermore, the chemokine receptor CX3CR1 has been reported to mark selective differentiation states of human and murine T cells^36^ suggesting that chemokine receptors, as markers of lineage differentiation, may have broader applicability.

We propose that the *Retnla+* population represents a premature macrophage population and that multiple iCCR expression equips these cells with the ability to rapidly migrate toward a range of possible pathogens, or tissue damage, potentially associated with alternative chemokine ligand expression. We further hypothesize that iCCR expression is then progressively restricted in the macrophages in response to sensing of the particular inflammatory environment, thus aligning iCCR expression with pathogen challenge. In addition, our data show that bacterial pathogen mimetics (i.e. TLR ligands) can skew macrophage iCCR expression in favor of CCR1 suggesting that final iCCR expression patterns can be manipulated by the inflammatory environment. In terms of CCR1, it is of note that CCL3 is more strongly induced by LPS than Poly(I:C), suggesting that this chemokine may be preferentially important for antibacterial responses. Importantly, CCL3 has been shown, in a range of inflammatory situations, to be a physiological substrate for the enzyme DPPIV (CD26).^37^ This enzyme (expressed in the lung: https://www.gtexportal.org/home/gene/DPP4) (Fig. S4C) cleaves the 2 most N-terminal amino acids from CCL3, changing its receptor-binding patterns from similar affinities for CCR1 and CCR5 to predominantly CCR1 binding.^38,39^ Thus, the ability of LPS to induce both CCR1 in macrophages, and CCL3 at inflamed sites, aligns macrophage chemotactic responsiveness to pathogen-specific chemokine expression. Further investigation is required to try to align alternative pathogen challenges with specific iCCR expression changes and associated ligand profiles.

Therefore, in summary, we have used transcriptomic approaches to demonstrate that inflammatory chemokine receptor expression in macrophages progresses from multireceptor expressing macrophages to more restricted expression in mature cells. Our data therefore confirm multiple iCCR expression on macrophages and demonstrate that this is not a consequence of redundancy, but rather that individual receptor expression patterns delineate transcriptionally distinct cells on the monocyte to mature-macrophage trajectory.

## Supplementary Material

qiag059_Supplementary_Data

## Data Availability

Raw and processed sequencing data have been uploaded to Gene Expression Omnibus. scRNA-seq data has the accession number: GSE305101 and bulk RNA-seq data have the accession number: GSE306514. Original code used to analyze scRNA-seq data was uploaded to Zenodo and can be found at the following: https://doi.org/10.5281/zenodo.16876331
