# Supplementary material for: Chemokine receptor expression defines a trajectory from monocytes to mature macrophages in the lung
Source: J Leukoc Biol. 2026 May 6;118(5):qiag059. doi: 10.1093/jleuko/qiag059 (PMC13196878; doi:10.1093/jleuko/qiag059)
Supplement: qiag059_Supplementary_Data [file qiag059_supplementary_data.docx]

**Supplementary data**

Chemokine receptor expression defines a trajectory from monocytes to mature macrophages in the lung. Mathie et al.


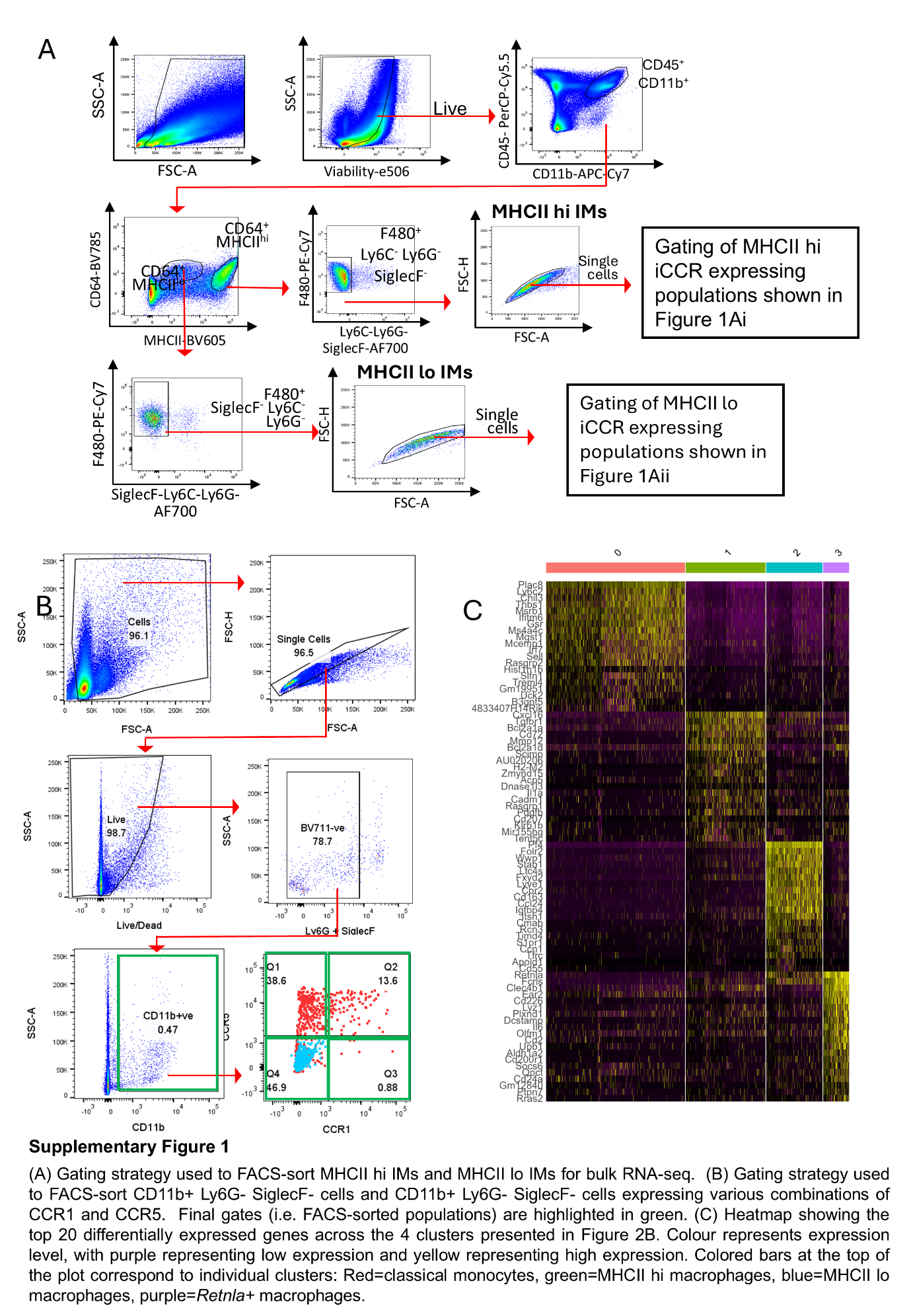


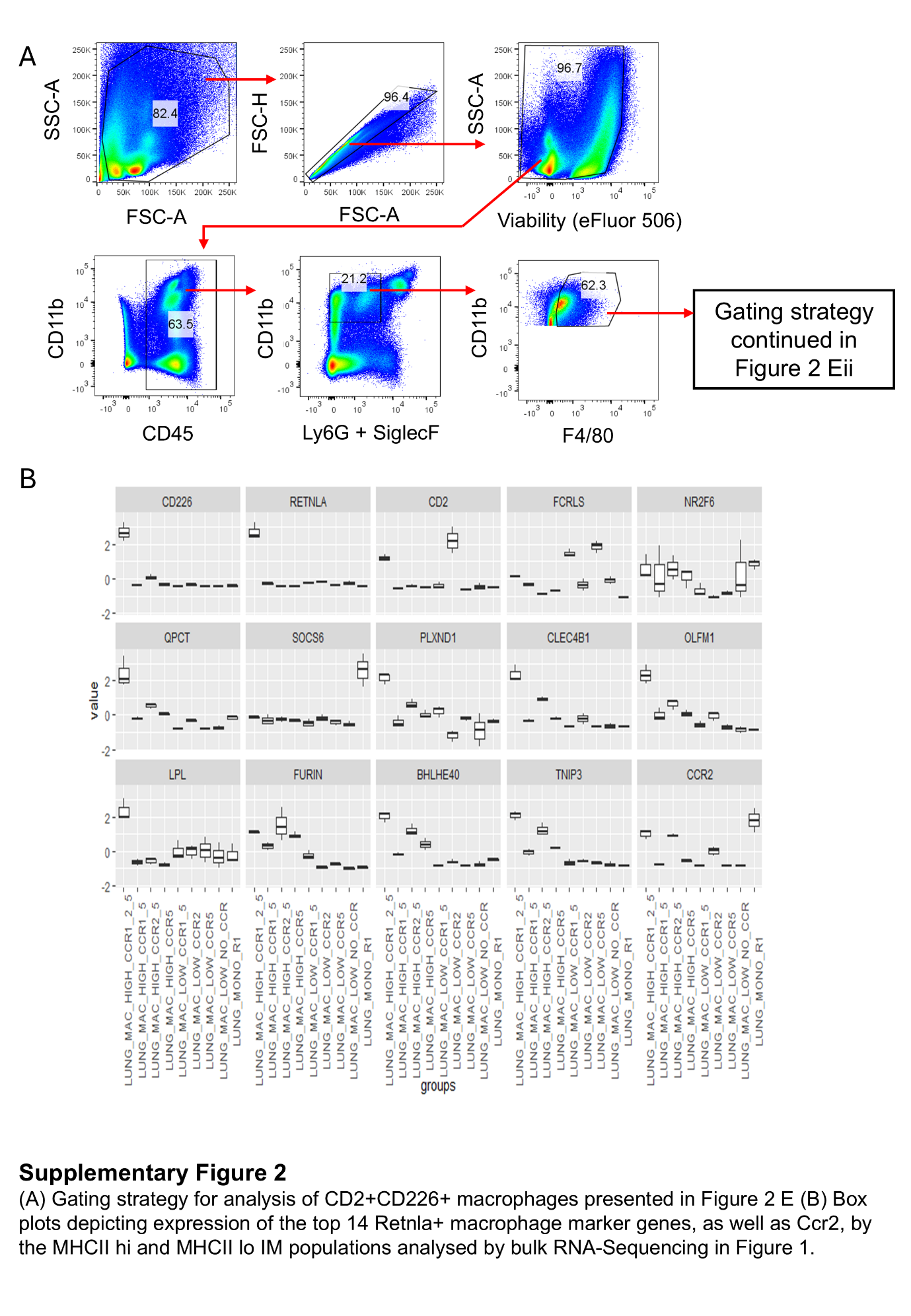


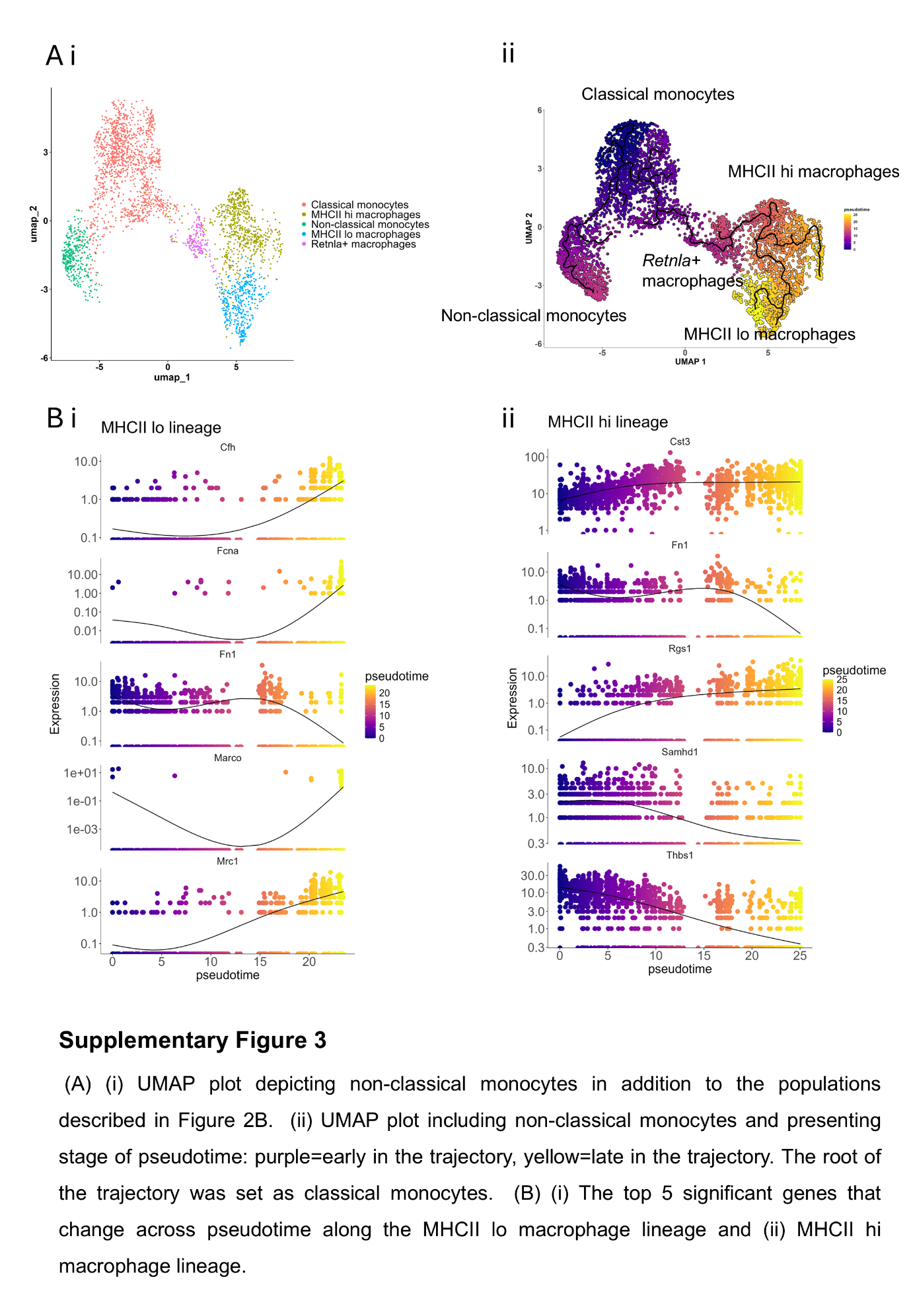


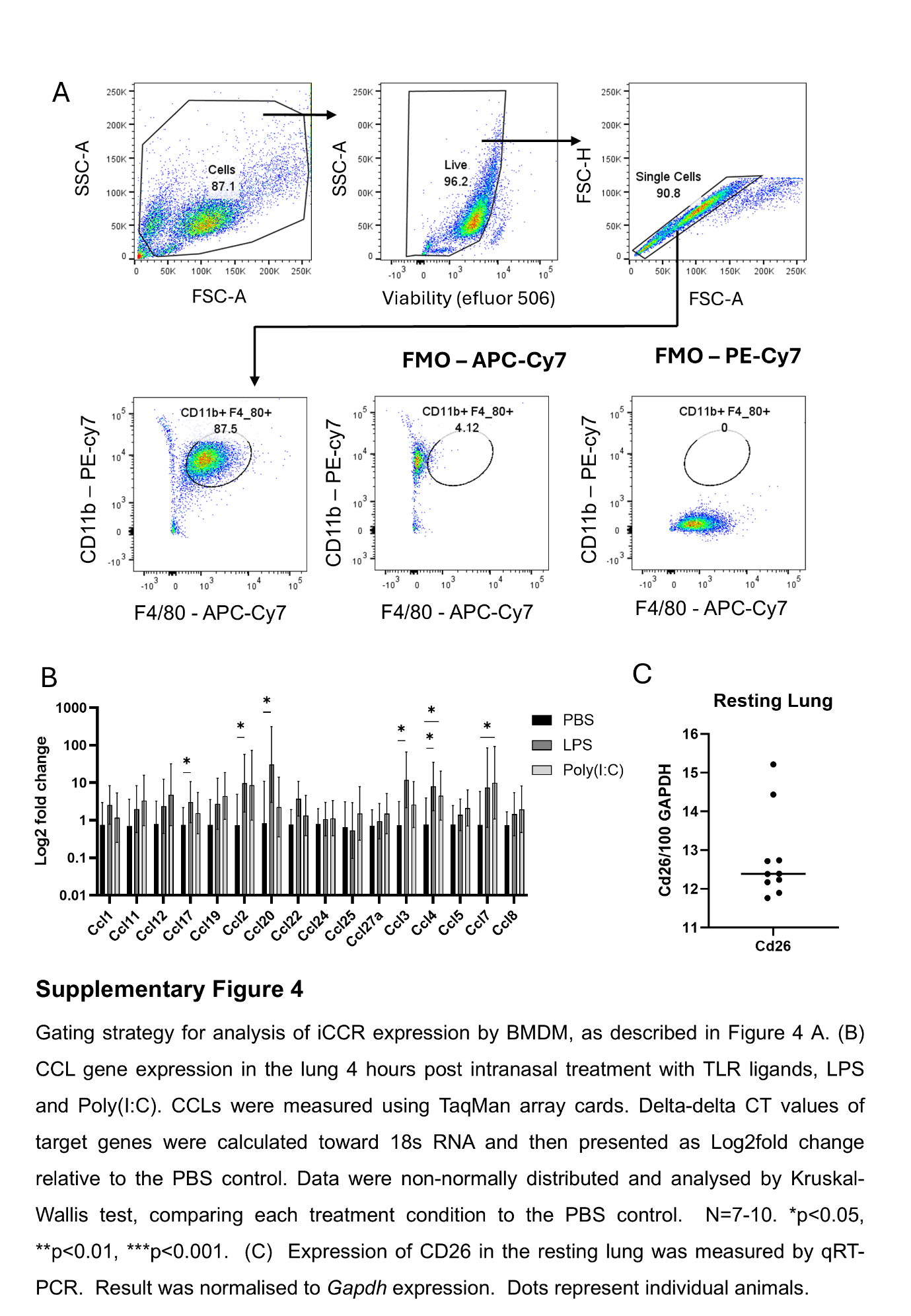


**Supplementary Table Legends.**

**Supplementary Table 1:** list of genes differentially expressed between the 16 clusters depicted in Figure 2A.

**Supplementary Table 2:** list of genes differentially expressed between the monocyte and macrophage clusters depicted in Figure 2B.
